# The cystogenic effects of ouabain in autosomal dominant polycystic kidney disease require cell caveolae

**DOI:** 10.1016/j.yexcr.2024.114356

**Published:** 2024-11-23

**Authors:** Jordan Trant, Gladis Sanchez, Jeffery P. McDermott, Gustavo Blanco

**Affiliations:** Department of Cell Biology and Physiology and the Kidney Institute, University of Kansas Medical Center, Kansas City, KS, USA

**Keywords:** Na,K-ATPase, Caveolae, Autosomal dominant polycystic kidney disease

## Abstract

We have previously shown that the hormone ouabain is a circulating factor which can accelerate the progression of autosomal dominant polycystic kidney disease (ADPKD). At physiologic concentrations, ouabain increases cyst area and fibrosis in kidneys from ADPKD but not wildtype mice. These effects are due to an increased affinity for ouabain by its receptor, Na,K-ATPase (NKA), in the kidneys of ADPKD mice which leads to over-activation of NKA signaling function. Previous studies suggested that ouabain’s stimulation of NKA signal transduction is mediated by NKA located within cell caveolae. Here, we determined whether caveolae are involved in the ouabain-induced progression of ADPKD cysts. We generated an ADPKD mouse with a global knockout of the main structural component of caveolae, caveolin-1 (CAV1), which we confirmed lacks caveolae in the kidney. When given physiological amounts of ouabain for 5 months, *Pkd1*^*RC/RC*^*Cav1*^−/−^ mice did not exhibit any changes in cyst progression, contrasting with the *Pkd1*^*RC/RC*^ mice which showed a significant increase in cystic area and kidney fibrosis. Also, measures of ouabain-induced cell proliferation, including the number of Ki67-positive nuclei and phosphorylation of the extracellular regulated kinase (ERK) and protein kinase B (Akt), did not increase in the *Pkd1*^*RC/RC*^*Cav1*^−/−^ mice compared with the *Pkd1*^*RC/RC*^ mice. Moreover, the abnormally increased affinity for ouabain of NKA in *Pkd1*^*RC/RC*^ mice was restored to wildtype levels in the *Pkd1*^*RC/RC*^*Cav1*^−/−^ mice. This work highlights the role of caveolae in ouabain-induced NKA signaling and ADPKD cyst progression.

## 1. Introduction

Autosomal dominant polycystic kidney disease (ADPKD) is the most common monogenetic disorder of the kidney, present in at least 1:1000 live births worldwide [1–3]. The disease affects roughly half a million people in the US and has an estimated economic burden of over $7 billion annually [4]. ADPKD is characterized by the formation of fluid-filled cysts in the kidneys, a process which has been shown to begin *in utero* and then continue to grow throughout the lifetime of the affected patients [5]. Eventually, cysts get large enough to compress the normal, functional areas of the kidney, reducing kidney function and causing renal failure. In addition to these symptoms, patients can often also have cysts in the liver and pancreas, vascular malformations, and aneurysms, all of which contribute to lower quality and quantity of life [6].

ADPKD is caused by mutations in *PKD1* or *PKD2* genes which encode the proteins polycystin-1 and polycystin-2, respectively. It is well established that in addition to the germline mutation in *PKD1* or *PKD2*, a second or even third hit is necessary to cause cyst formation and growth [7,8]. The exact roles of polycystin-1 and polycystin-2 in the kidney are still under investigation, but it is known that these proteins associate to form a heteromeric complex which functions as a nonspecific calcium channel that influences cell proliferation, adhesion, migration, and maintain the normal renal epithelial architecture and function [9–13]. Interestingly, the progression and kidney damage caused by cysts in ADPKD is highly variable, even among patients with the same mutation, suggesting a substantial environmental role in ADPKD pathogenesis [5]. Specifically, several factors which circulate in the blood and influence ADPKD have been found, including the hormone ouabain which our lab identified as a substance which accelerates ADPKD progression [14,15]. Ouabain and other cardenolide steroidal molecules are present in the human circulation in nanomolar concentrations and are produced endogenously as hormones or incorporated through plant sources in the diet [16–18]. Using different *in vitro* approaches, we have shown that physiologic doses of ouabain stimulate cell proliferation, induce epithelial to mesenchymal transition (EMT), and activate cAMP-dependent fluid secretion in cells obtained from kidney cysts of patients with ADPKD while having none of these effects in normal human kidney cells [19–21]. More recently, we have shown that ouabain increases ADPKD cyst progression and kidney fibrosis *in vivo* in the slowly progressive ADPKD mouse model, *Pkd1*^*RC/RC*^ [22].

Ouabain’s actions require it to bind to its specific receptor, the Na,K-ATPase (NKA) [23]. NKA is an integral plasma membrane protein which works as an active ion transport system that uses the energy from ATP hydrolysis to move Na^+^ out of and K^+^ into the cell [24]. In addition to its role in maintaining ion gradients, NKA also functions as the receptor and signal transducer that mediates the effects of ouabain in cells [25]. Once bound to ouabain, NKA recruits and activates intracellular messengers and stimulates downstream phosphorylation of effector proteins, resulting in the regulation of proliferation, metabolism, motility, and adhesion in a cell type- and tissue-specific manner [26–28]. Specifically, NKA can activate Src kinase, one of the main transducers of ouabain-induced NKA signaling, but has also been shown to increase reactive oxygen species (ROS) signaling, IP3 receptor signaling, and activate mTOR/Akt signaling, among others [29–34]. These effects of ouabain are concentration dependent, as high concentrations of ouabain can be toxic through the inhibition of NKA ion transport.

NKA exists as two different pools: one in the general plasma membrane, which in the renal epithelium is distributed along the basolateral membrane of the cells, and the other confined to cell caveolae [31,35]. Caveolae are flask-shaped, specialized microdomains of the plasma membrane which can serve as the center for the scaffolding of a series of cell signaling receptors and mediators, including the epithelial nitric oxide synthase (eNOS), epithelial growth factor receptor (EGFR), the insulin receptor, and NKA [36–38]. Caveolae require cholesterol and members of caveolin and cavin protein families to form and are present in several tissue types, including striated muscle, adipose, and the renal epithelial cells of the distal nephron [39]. There is evidence in cardiac and renal cell lines that the NKA involved in ouabain-stimulated signaling reside mostly within the caveolae, which led to the idea that caveolae may be a hub for NKA signaling [38,40].

We have previously found that the effects of ouabain in ADPKD both *in vitro* and *in vivo* are due to an overactivation of NKA signal transduction at physiological ouabain concentrations which accelerates progression of the disease. This is due to an increased affinity for ouabain exhibited by a fraction of the total NKA of human ADPKD cells as well as kidneys isolated from a slowly progressive ADPKD mouse model, *Pkd1*^*RC/RC*^, which causes an enhanced response to circulating concentrations of ouabain [19,22]. This increased response promotes several hallmarks of ADPKD cystogenesis, such as cell proliferation, cell dedifferentiation, and transepithelial fluid secretion. In addition, we found that *Pkd1*^*RC/RC*^ mice expressing a mutated form of NKA with high affinity for ouabain have dramatically increased cyst progression, supporting the role of NKA ouabain affinity in ADPKD progression [22].

In this work, we investigated the role of caveolae in NKA signaling in ADPKD *in vivo* through a mouse in which caveolar formation is disrupted on the ADPKD background. This model was obtained by crossing a knockout mouse model of caveolin-1 (CAV1), a main structural component required for caveolae formation, and the slowly progressive ADPKD mouse line, *Pkd1*^*RC/RC*^ [41,42]. The resulting *Pkd1*^*RC/RC*^*Cav1*^−/−^ model does not form caveolae in the kidney, lacks the abnormal high ouabain affinity component of NKA, and no longer responds to the pro-cystogenic effects of ouabain typically observed in *Pkd1*^*RC/RC*^ mice. Overall, this model supports the idea that caveolae are necessary for the exacerbated ouabain-induced NKA signaling that contributes to ADPKD progression.

## 2. Materials and methods

### 2.1. Generation of mouse models

All experimental protocols in this work involving mice were approved by the University of Kansas Medical Center Institutional Animal Care and Use Committee. The *Pkd1*^*RC/RC*^ mice were originally obtained from the University of Kansas Medical Center (KUMC) Rodent Models & Drug Testing Core. Genotyping for this mouse was performed according to Hopp et al. [43]. The *Cav1*−/− mice were originally created by Razani et al. [41] and were obtained from the Jackson Laboratory (Bar Harbor, ME) strain #007083. Genotyping for this mouse was performed as described in the originating publication. We crossed this mouse with *Pkd1RC/RC* mice to obtain a global caveolin-1 knockout under the ADPKD genotype (*Pkd1RC/RCCav1*−/−).

### 2.2. Reverse transcription polymerase chain reaction (RT-PCR) for CAV1

Total kidney RNA was prepared using the TRIzol reagent following the supplier specifications (Life Technologies). A total of 10.0 mg RNA was treated with RNase-free DNase I (Roche, Indianapolis, IN). The prep was further cleaned up with the RNeasy Mini Kit (Qiagen) following the manufactures instructions. Complementary DNA was prepared by reverse transcription of 1.0 mg of total RNA using the cDNA Synthesis SuperMix kit (Bimake). The resulting PCR amplified fragment used primers specific for mouse cav1 gene (NCBI Reference Sequence: NM_007616.5) using sense: 5’ - ATGTCTGGGGGCAAATACGTA and antisense: 5’ - GAAGCTCTTGATGCACGGTAC. RNA quality assurance was checked with primers specific to mouse beta actin (NCBI Reference Sequence: using sense: 5’ - CCGTGAAAAGATGACCCAG and antisense: 5’ - TAGCCACGCTCGGTCAGG). Amplified DNA fragments were identified by electrophoresis in 1 % agarose gels stained with ethidium bromide. Expected sizes for the amplification are 411 and 249 bp, respectively.

### 2.3. Electron microscopy analysis of caveolae

*Pkd1*^*RC/RC*^ and *Pkd1*^*RC/RC*^*Cav1*^−/−^ animals were sacrificed by carbon dioxide per KUMC IACUC guidelines prior to organ harvest. Immediately after sacrifice, kidneys were injected with 2.5 % glutaraldehyde in 1M cacodylate buffered fixative in several places with a 21G needle. Kidneys were isolated and cleaned on ice on a drop of fixative. Kidneys were shaved to sizes roughly 1 × 3 × 5 mm and fixed overnight on a rotator at 4 °C in 2.5 % glutaraldehyde in 1M cacodylate buffered fixative. Sections were mounted, prepared, and imaged by the KUMC Electron Microscopy Research Laboratory (Kansas City, KS) using the JEOL JEM-1400 Transmission Electron Microscope equipped with a Lab6 gun. This facility is a part of the Kansas Intellectual and Developmental Disabilities Research Center (KIDDRC) supported by KIDDRC NIH U54 HD 090216 (Kansas City, KS).

### 2.4. Ouabain treatment protocols for mice

Mice were randomly assigned to receive injections of ouabain dissolved in saline solution or saline alone. Ouabain solution was made fresh every 14 days and kept at 0.03 μg/μL. *Pkd1RC/RC* and *Pkd1RC/RCCav1*−/− mice received a daily dosage of 0.3 μg/g body weight. Mice were injected via intraperitoneal injection daily from day 8 after birth until 5 months of age. At that point, mice were euthanized, and the kidneys were preserved for the study.

### 2.5. Histological analysis of kidneys

Kidneys were analyzed from mice after 5 months of injections of saline or ouabain. Animals were sacrificed by carbon dioxide per KUMC IACUC guidelines prior to organ harvest. Kidneys were isolated, weighed, and photographed before being fixed in Bouin’s solution for 16 h at 4 °C on a rotator. After this time, kidneys were washed 3 times for 5 min in 70 % ethanol, then stored in fresh 70 % ethanol until processing. Tissue section and hematoxylin/eosin (H&E) staining was completed by the Histology Core Facility of the Kansas Intellectual and Developmental Disabilities Research Center (KIDDRC) supported by KIDDRC NIH U54 HD 090216 (Kansas City, KS). Slides were imaged using a 1x objective on a Nikon 80i microscope. Where sections exceeded the visual field, images were photomerged using Adobe Photoshop software. Quantification of total kidney area and percent cyst area of each section was calculated using ImageJ 1.53k software. Fractional cyst area was calculated as the ratio of the total dilated cystic tubule area divided by the area of the whole kidney.

### 2.6. Immunofluorescence of kidney sections

Unstained kidney sections prepared by the KUMC Histology Core were rehydrated in sequential washes of xylenes (2 × 5 min) and 100 %, 95 %, 70 %, 50 %, and 30 % ethanol in saline (5 min each), then rinsed in water for 5 min. Antigen retrieval was performed with citrate buffer (pH 6.0) and microwaved for 15 min. Then, sections were quenched for 30 min with 0.1 M ammonium chloride in saline then permeabilized for 5 min with 0.1 % Triton X-100 in saline (for Ki67) or 0.2 % Tween in saline for 15 min (for CAV1 and NKAα1). Sections were then blocked with 2 % BSA in saline for 60 min. Primary antibodies in 2 % BSA in saline were applied for 16hr at 4 °C in a hydration chamber. After 3 5-min saline washes, secondary antibodies were applied for 1 h in the dark in a hydration chamber. Slides were mounted with ProLong Gold Antifade with DAPI (ThermoFisher Scientific, Waltham, MA). Slides were imaged using 20x and 60x objectives on a Nikon 80i fluorescence microscope. Antibodies used for immunofluorescence include: Caveolin-1 at 1:200 (MA3–600 from ThermoFisher, Waltham, MA); Ki67 at 1:1000 (15580 from Abcam, Waltham, MA); NKAα1 at 1:1000 (14418–1-AP from Proteintech, Rosemont, IL); Invitrogen goat anti-mouse and goat anti-rabbit Alexafluor 488 or 594 secondary antibodies at 1:1000 (ThermoFisher, Waltham, MA).

### 2.7. Determination of Ki67-positive nuclei

After staining for Ki67 with a DAPI counterstain as mentioned above, 12–15 cystic regions of each kidney tissue section were examined for Ki-67 positive nuclei. Cystic regions were randomly selected through a UV Excitation 360/40, Emission 460/50 filter to visualize all nuclei under the DAPI stain. Images of all nuclei were imaged first with a 20X objective, followed by the Ki-67 positive nuclei. The number of Ki67-positive nuclei and the total number of nuclei within one region was determined using ImageJ as described [44]. 12–15 regions per tissue section were averaged to get mean number of Ki67-positive nuclei per 1000 nuclei per tissue section. 4 animals per group, mixed male and female, were examined.

### 2.8. Determination of kidney fibrosis

Picrosirius Red staining was used in order to detect fibrosis and collagen deposition on kidney sections, following previously described protocols [45]. Quantification of the extent of fibrosis was determined using ImageJ 1.53k software.

### 2.9. Kidney membrane preparation

To obtain a crude preparation of membranes, kidneys were minced with a razor blade then homogenized in a glass homogenizer using 40 strokes in a Suspension Buffer containing: 250 mM sucrose, 25 mM imidazole pH 7.4, 0.1 mM EGTA, 0.5x cOmplete mini Protease Inhibitor Cocktail (Roche, Indianapolis, IN). The solution was then centrifuged at 1000×*g* for 5 min at 4 °C. The supernatant was collected, and the pellet was resuspended in 1.0 mL of Suspension Buffer and homogenized with 40 strokes in a glass homogenizer and centrifuged again as described above. The supernatants from both centrifugations were combined and spun at 30,000×*g* for 30 min in a Beckman TLA-55 rotor at 4 °C (Beckman Coulter, Indianapolis, IN). The pellet was resuspended in 1.0 mL of Suspension Buffer. Protein concentration of the sample was measured using the dye-binding assay based on the method of Bradford, from Bio-Rad (Hercules, CA, USA). Samples were adjusted to 1.0 mg/mL protein and BSA and sodium deoxycholate were added at 0.75 μg/μg protein and 0.1 % final concentration, respectively. After mixing for 30 min, the solution was ultracentrifuged at 100,000×*g* for 60 min at 4 °C (Beckman TLA-55 rotor). The supernatant was discarded, and the pellet was resuspended in 200 μL of Suspension Buffer to be used in the assay.

### 2.10. Na,K-ATPase activity assays

Na,K-ATPase activity was determined by the initial rate of release of Pi from ATP, using malachite green as described previously [46]. The assay was adapted to a microtiter plate format with each well of a 96-well plate containing a final volume of 100 μl. Briefly, the incubation solution contained: 120 mM NaCl, 30 mM KCl, 3 mM MgCl2, 0.2 mM EGTA and 30 mM Tris–Cl, pH 7.4. The assay was performed in the absence or presence of different concentrations of ouabain. A total of 0.125 μg of membrane protein was used for each sample. After a 5-min preincubation at 37 °C, the reaction was started by the addition of ATP to a final concentration of 1 mM. Assay was performed for 30 min at 37 °C and stopped by addition of the Malachite Green dye. The specific total NKA activity was defined as the Na^+^ and K^+^ dependent ATP hydrolysis sensitive to 10^−3^ M ouabain [47]. Dose-response curves for the ouabain inhibition of NKA activity were fitted using a monophasic or a biphasic model, representing a single or two populations of NKA with different affinities for ouabain, as described [47].

### 2.11. Immunoblot analysis

Whole kidneys were isolated from mice and homogenized in a buffer containing 250 mM sucrose, 25 mM imidazole pH 7.4, 0.1 mM EGTA, 0.5x Roche cOmplete mini Protease Inhibitor Cocktail, and 0.5x Roche PhosSTOP Phosphatase Inhibitor Cocktail (Sigma, St. Louis, MO) on ice. Protein concentrations were determined using the dye-binding assay from Bio-Rad (Hercules, CA). 50 μg of protein from each sample were separated by 10 % SDS-PAGE and transferred onto PVDF membranes (ThermoFisher, Waltham, MA). The antibodies used for immunochemistry include those against phospho-p44/42 (ERK1/2), p44/42 (ERK1/2), pAkt, AKT, and α-tubulin (from Cell Signaling; Danvers, MA); and against collagen-I (from Abcam; Waltham, MA). Horseradish peroxidase-conjugated secondary antibodies (from Jackson ImmunoResearch; West Grove, PA) and chemiluminescence were used for detection. Images were developed and captured by ChemiDoc MP from BioRad (Hercules, CA) and analyzed using ImageJ 1.53k software. The level of phosphorylation of proteins was determined as the ratio of the phosphorylation of the protein to the total form of the protein expressed as relative density, then relative to *Pkd1RC/RC* control amounts.

### 2.12. Statistical analysis

Statistical significance of the differences between means was determined by a two-way ANOVA with Holm-Šídák correction for multiple comparisons using GraphPad Prism 9.0.0. Statistical significance was defined as adjusted P < 0.05 after multiple corrections.

## 3. Results

### 3.1. Pkd1^RC/RC^Cav1^−/−^ mice do not express caveolin-1 or form caveolae in the kidney

**Fig. 1.**
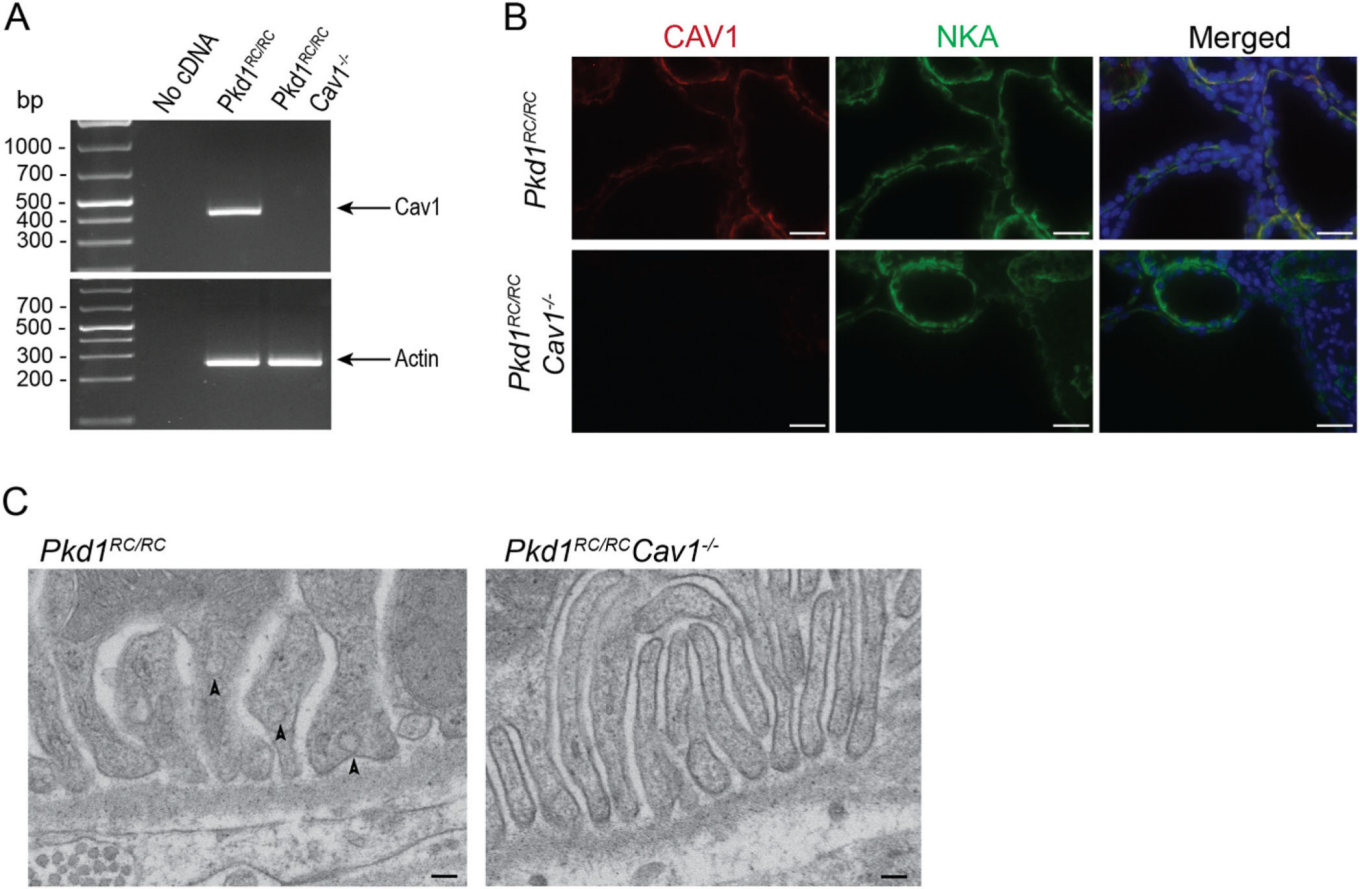
Characterization of *Pkd1*^*RC/RC*^*Cav1*^−/−^ mouse model. (A) Polymerase chain reaction (PCR) demonstrating the presence and absence of caveolin-1 cDNA in *Pkd1*^*RC/RC*^ and *Pkd1*^*RC/RC*^*Cav1*^−/−^ mice, respectively. (B) Immunofluorescence of CAV1 and NKA staining in the kidney tubules from *Pkd1*^*RC/RC*^ (top row) and *Pkd1*^*RC/RC*^*Cav1*^−/−^ (bottom row) mice. Scale bar represents 50 μm. (C) Transmission electron microscopy demonstrating the presence and absence of caveolae in kidney tubules in *Pkd1*^*RC/RC*^ (left) and *Pkd1*^*RC/RC*^*Cav1*^−/−^ (right) mice, respectively. Photomicrographs are from collecting duct cells from kidneys from each model. Scale bar represents 100 nm.

To determine the involvement of caveolae in ouabain-induced NKA signaling in ADPKD mice, we crossed a global caveolin-1 knockout mice into the *Pkd1*^*RC/RC*^ mouse line to generate *Pkd1*^*RC/RC*^*Cav1*^−/−^ mice. Like the lines from which it originated, this model is viable and fertile. We validated the absence of CAV1 expression in the kidneys of this model, as shown by the lack of CAV1 mRNA and protein (Fig. 1A and B). We also determined that the expression of NKA was normal and localized to the basolateral side of the epithelial tubular cells of both *Pkd1*^*RC/RC*^ and *Pkd1*^*RC/RC*^*Cav1*^−/−^ kidneys (Fig. 1B). In the kidney tubules, NKA and CAV1 colocalized, as has been previously shown in wild type mice [48]. Though it has been shown that a loss of CAV1 disrupts caveolar formation in renal tubular cells [42,49], we verified this in our mouse model using transmission electron microscopy. As shown in Fig. 1C, *Pkd1*^*RC/RC*^ kidney tubules present caveolae normally along the basolateral membrane of collecting duct cells. As expected, the renal tubular epithelium of *Pkd1*^*RC/RC*^*Cav1*^−/−^ mice demonstrate no caveolar formation (Fig. 1C). Therefore, CAV1 is essential for caveolar formation in the kidneys of *Pkd1*^*RC/RC*^ mice, and its removal causes caveolar disruption.

### 3.2. NKA ouabain affinity reverts to normal with the loss of caveolae in Pkd1^RC/RC^ mice

**Figure 2.**
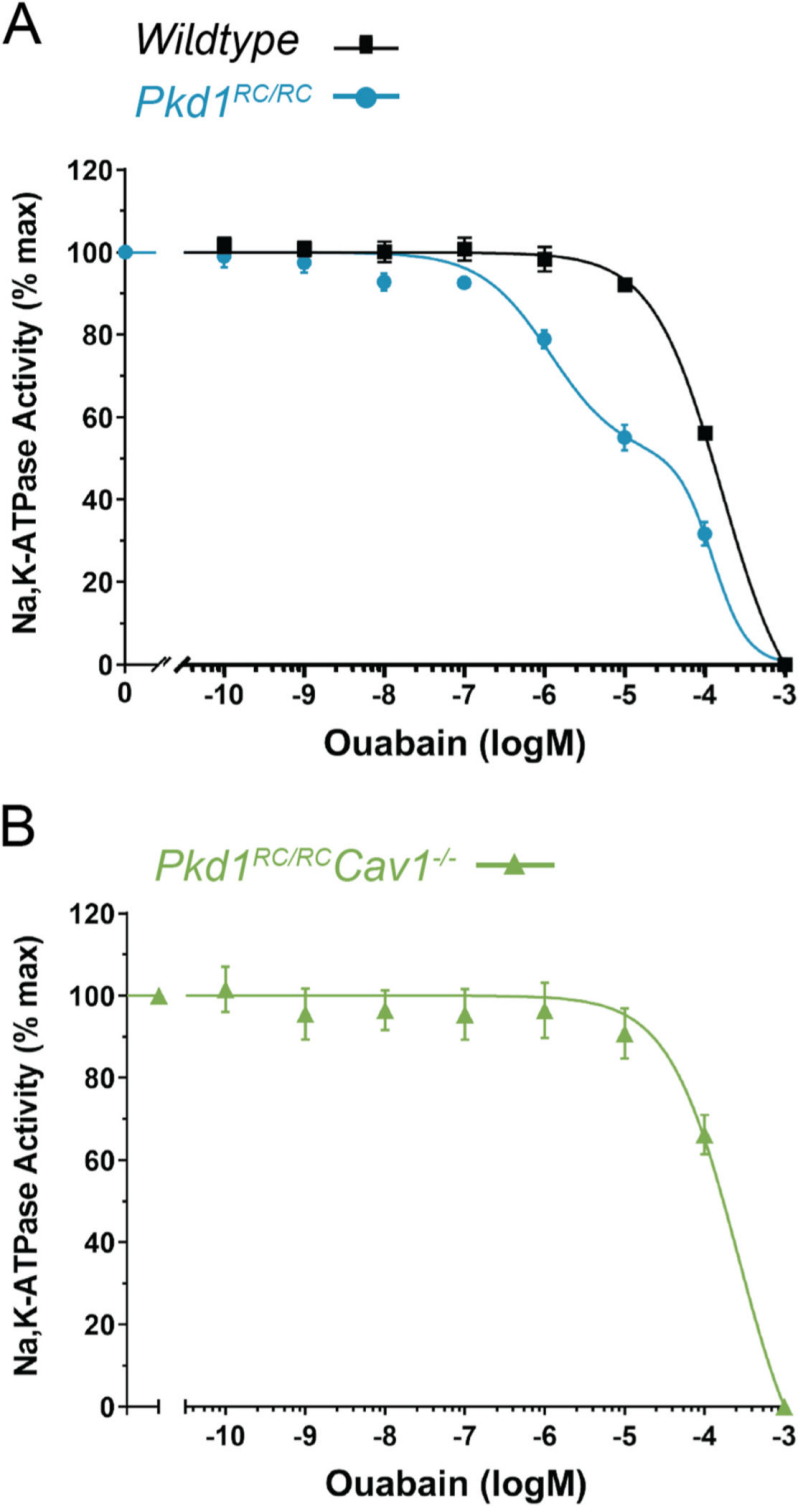
CAV1 knockout abolishes the abnormal ouabain affinity component of NKA in *Pkd1*^*RC/RC*^ mouse kidneys. (A) Ouabain affinity of NKA in WT (black squares, black curve) and *Pkd1*^*RC/RC*^ (blue circles, blue curve) mouse kidney homogenates. (B) Ouabain affinity of NKA in *Pkd1*^*RC/RC*^*Cav1*^−/−^ (green triangles, green curve) mouse kidney homogenates. *Pkd1*^*RC/RC*^ mouse kidneys exhibit a biphasic dose response to ouabain with a low- and high-affinity response. WT and *Pkd1*^*RC/RC*^*Cav1*^−/−^ mouse kidneys show a monophasic curve with a relatively low affinity for ouabain which have similar IC_50_. Data for panel (A) is reformatted for comparison from a previous publication [22].

We have previously shown that the primary driver of the cystogenic effects of ouabain in *Pkd1*^*RC/RC*^ mice and human ADPKD cells is the abnormally high affinity that a fraction of NKA has for ouabain. Normally, the NKA ouabain affinity of the wild type mouse kidney is relatively low, with an IC_50_ value of 2 × 10^−4^ M [22]. However, in *Pkd1*^*RC/RC*^ mice, a biphasic inhibition response for ouabain is found. Thus, ~50 % of the Na,K-ATPase has a low affinity for ouabain similar to that of wild type kidneys (IC_50_ of 1.21 ± 0.38 × 10^−4^ M), but the remaining 50 % presents an affinity that is roughly 2–3 orders of magnitude higher, with a calculated IC_50_ of 1.26 ± 0.19 × 10^−6^ M (Fig. 2A) [22]. This high affinity pool of NKA allows the low circulating levels of ouabain to persistently stimulate NKA signal transduction and to drive pro-cystic pathways in cystic cells [22]. Studies performed on the ouabain effects in the heart have suggested that NKA signaling occurs mostly within the caveolar microdomains of the plasma membrane, where NKA associates with a series of signaling partner molecules [37]. Here, we determined whether abrogating the formation of cell caveolae affects the binding capacity of the NKA receptor to ouabain. To achieve this, we performed dose response curves for the ouabain inhibition of NKA in kidney membrane fractions from *Pkd1*^*RC/RC*^*Cav1*^−/−^ kidneys. Ablation of caveolae in ADPKD mouse kidneys resulted in a change of the biphasic response to ouabain to monophasic, presenting a calculated IC_50_ value of 2.62 ± 1.17 × 10^−4^ M (Fig. 2B). This is similar to the IC_50_ for ouabain inhibition of wildtype mouse kidney [22] (Fig. 2A, black curve). These results show that preventing caveolar formation abolishes the high ouabain affinity component of the NKA of ADPKD cells, bringing the NKA response to ouabain back to the wildtype state.

### 3.3. Ouabain stimulates cyst progression in Pkd1^RC/RC^ but not Pkd1^RC/RC^Cav1^−/−^ mice

To determine whether caveolae are essential for ouabain to increase ADPKD cyst progression, we applied ouabain in normal saline or saline alone daily by intraperitoneal injection to *Pkd1*^*RC/RC*^ and *Pkd1*^*RC/RC*^*Cav1*^−/−^ mice from postnatal day 8 until 5 months of age. We used ouabain at 0.3 mg/kg body weight to maintain constant blood levels of ouabain within the physiological range as previously described [50–52]. At 5 months, mice were sacrificed, kidneys were dissected, weighed, photographed, fixed, sectioned, and stained with H&E and picrosirius red to determine cystic area and fibrosis, respectively.

**Fig.3.**
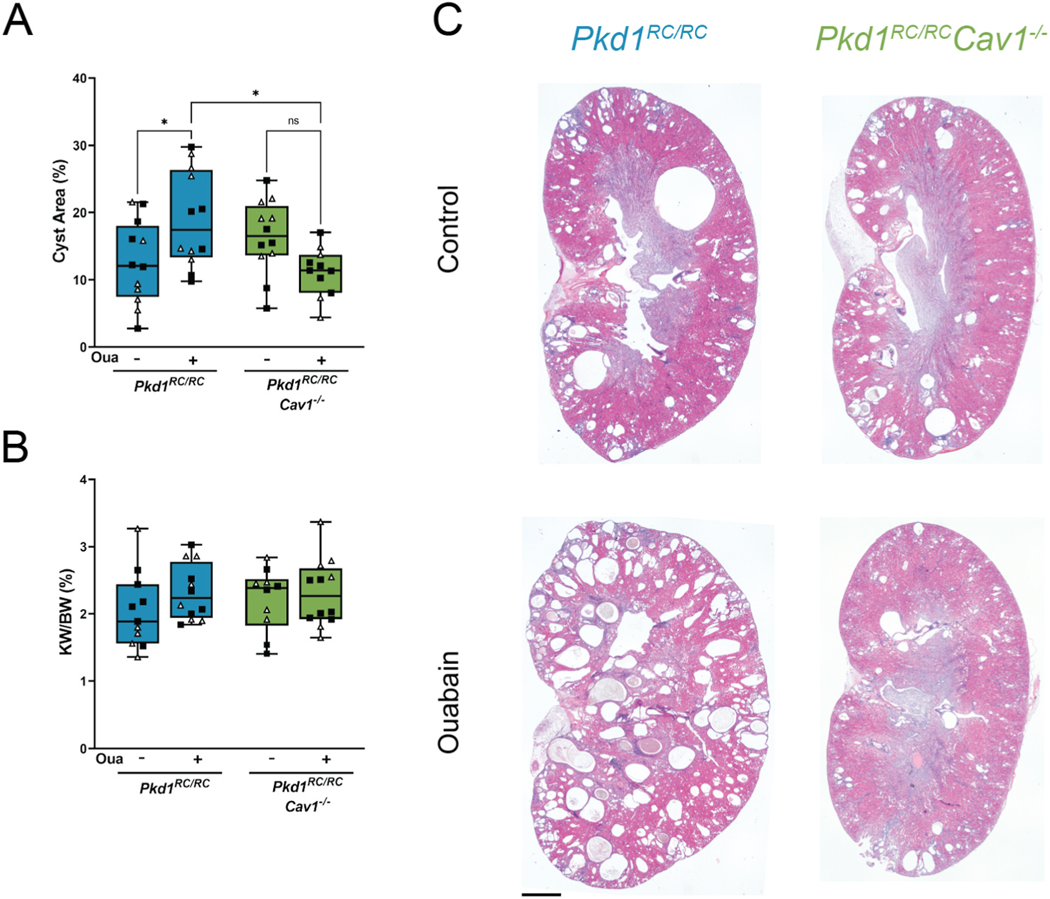
Ouabain is unable to increase cyst development and growth in *Pkd1*^*RC/RC*^*Cav1*^−/−^ mice. (A) Cystic index of *Pkd1*^*RC/RC*^ and *Pkd1*^*RC/RC*^*Cav1*^−/−^ mice. (B) Kidney weight to body weight ratio (KW/BW) of *Pkd1*^*RC/RC*^ and *Pkd1*^*RC/RC*^*Cav1*^−/−^ mice. (C) Representative kidney sections of *Pkd1*^*RC/RC*^ and *Pkd1*^*RC/RC*^*Cav1*^−/−^ mice stained with hematoxylin and eosin (H&E). Values are ± SEM of 12 animals per group, 6 male (black squares) and 6 female (white triangles). Scale bar represents 1000 μm *p < 0.05; **p < 0.01.

As shown in Fig. 3, the cystic area in the *Pkd1*^*RC/RC*^ mice was significantly increase after the administration of ouabain. This agreed with our previous results using this animal model [22]. By contrast, when compared to the *Pkd1*^*RC/RC*^ mouse kidneys, *Pkd1*^*RC/RC*^*Cav1*^−/−^ kidneys had no change in cystic area after ouabain administration. As we’ve previously shown, the percent kidney weight to body weight (% KW/BW) was not altered with ouabain administration at 5 months in either model (Fig. 3B). This is likely due to the slowly progressive nature of the RC mutation, which may require longer times for the kidney to achieve a higher mass [43]. Representative sections stained with H&E from *Pkd1*^*RC/RC*^ and *Pkd1*^*RC/RC*^*Cav1*^−/−^ kidneys are shown in Fig. 3C. As shown, the absence of caveolae reduces ouabain induced cyst growth in the *Pkd1*^*RC/RC*^ mice.

### 3.4. Ouabain increases cell proliferation in Pkd1^RC/RC^ but not Pkd1^RC/RC^Cav1^−/−^ mice

**Fig. 4.**
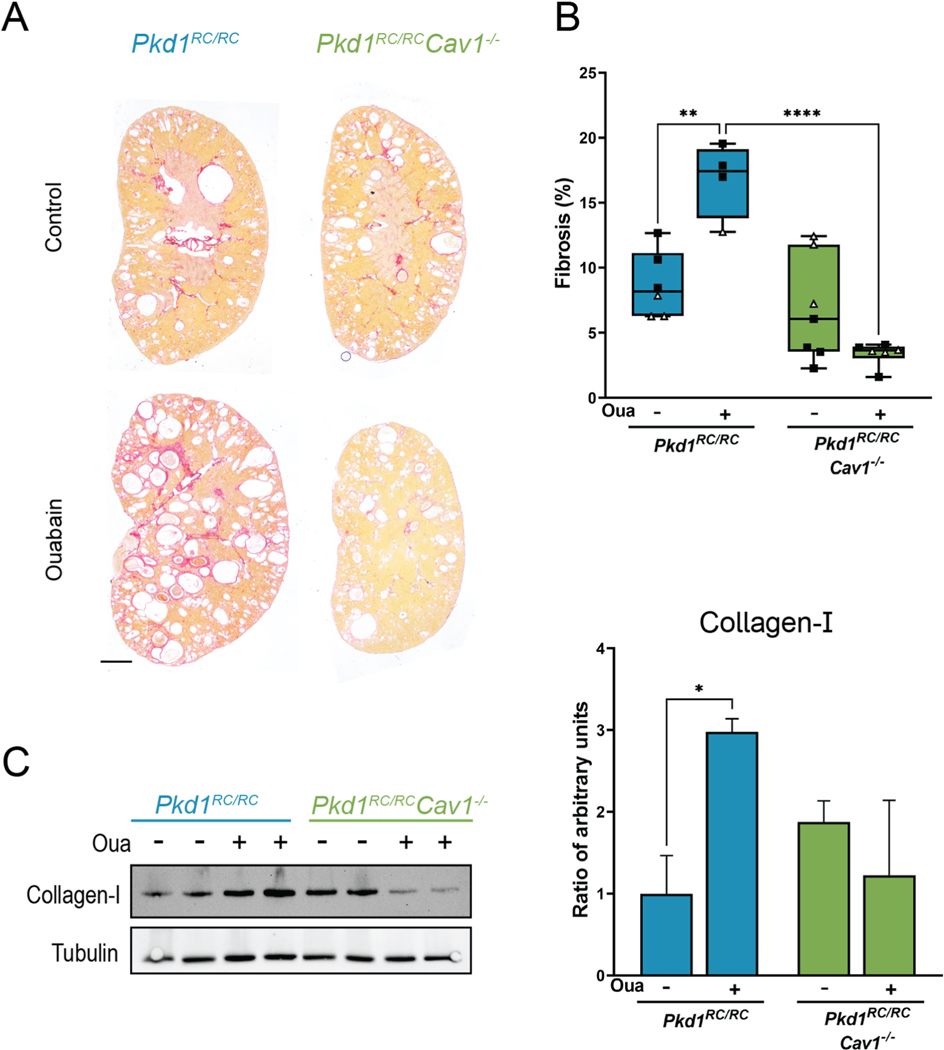
Ouabain increases cell proliferation in *Pkd1*^*RC/RC*^ but not *Pkd1*^*RC/RC*^*Cav1*^−/−^ mice. (A–B) Representative immunofluorescent images of total nuclei (top) and Ki67-positive nuclei (bottom) in *Pkd1*^*RC/RC*^ (A) and *Pkd1*^*RC/RC*^*Cav1*^−/−^ (B) mice. (C) Quantification of the average of total Ki67-positive nuclei from kidneys from both groups. Values are ±SEM of 4 animals per group, mixed male (black squares) and female (white triangles). *p < 0.05, ***p < 0.001.

We have previously shown that ouabain can increase cell proliferation in ADPKD cells [19]. To determine whether this process relies on the presence of caveolae, we used Ki67 as a marker for cell proliferation and counted the number of Ki67-positive nuclei per total nuclei in sections from *Pkd1*^*RC/RC*^ and *Pkd1*^*RC/RC*^*Cav1*^−/−^ mice with and without ouabain administration (Fig. 4A and B). As shown in Fig. 4C, ouabain significantly increased the number of Ki67-positive nuclei in *Pkd1*^*RC/RC*^ mice, which also agrees with the increased cystic area induced by ouabain in this model. In *Pkd1*^*RC/RC*^*Cav1*^−/−^ mice, however, no ouabain dependent change in the number of Ki67-positive nuclei was observed, with values similar to those of control *Pkd1*^*RC/RC*^ kidneys. This suggests that ouabain-induced cell proliferation in ADPKD requires the presence cell caveolae.

### 3.5. Ouabain increases fibrosis in Pkd1^RC/RC^ but not Pkd1^RC/RC^Cav1^−/−^ mice

**Fig. 5.**
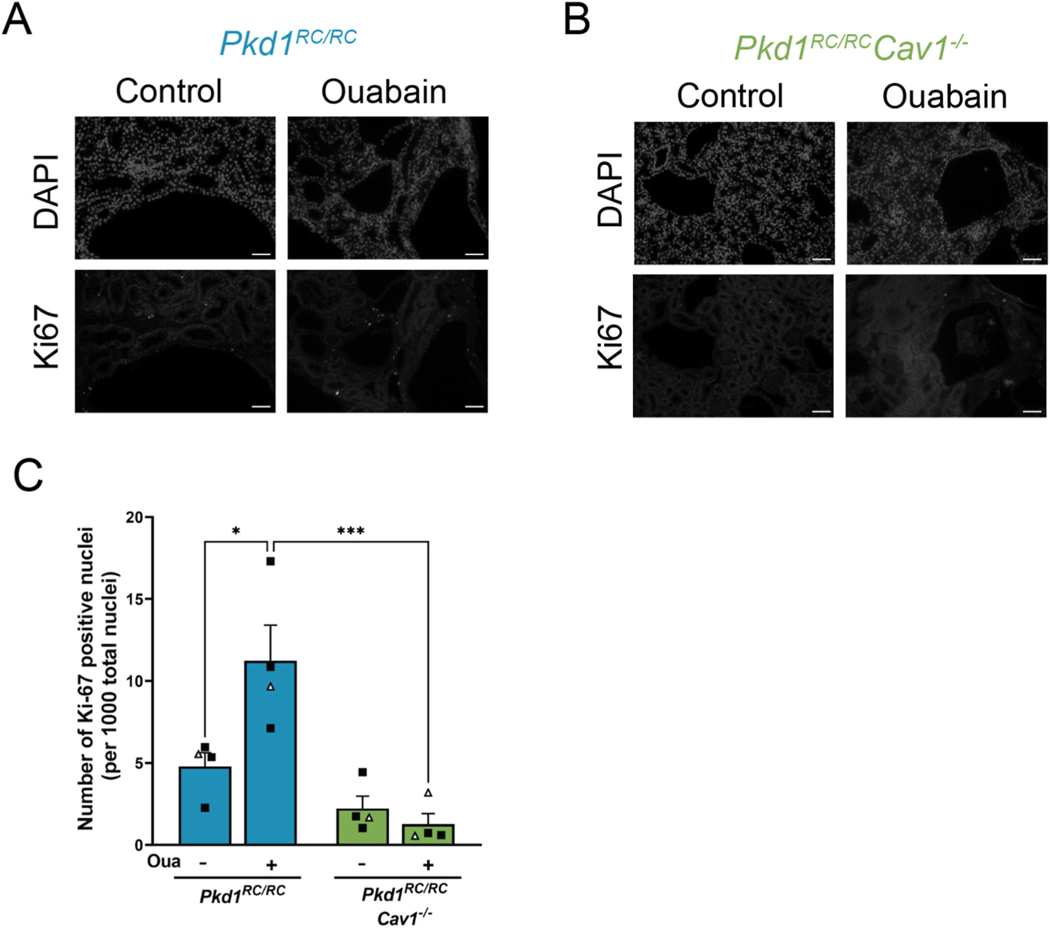
Ouabain is unable to increase fibrosis in *Pkd1*^*RC/RC*^*Cav1*^−/−^ mice. (A) Representative kidney sections of *Pkd1*^*RC/RC*^ and *Pkd1*^*RC/RC*^*Cav1*^−/−^ mice stained with picrosirius red. (B) Extent of kidney fibrosis in *Pkd1*^*RC/RC*^ and *Pkd1*^*RC/RC*^*Cav1*^−/−^ mice as determined by collagen deposition in the kidney. Values are ±SEM of 4–6 animals per group, mixed male (black squares) and female (white triangles). (C) Representative Western blots and quantification of collagen-I and tubulin. Values are ±SEM from 3 animals per group, mixed male and female. Scale bar represents 1000 μm *p < 0.05, ***p < 0.001.

We have previously demonstrated that ouabain increases epithelial-to-mesenchymal transition (EMT) in ADPKD cells, and that it enhances collagen deposition in *Pkd1*^*RC/RC*^ mouse kidneys [20,22]. Here, we examined whether caveolae are necessary to mediate ouabain-induced collagen deposition in *Pkd1*^*RC/RC*^ kidneys. As shown in Fig. 5, ouabain increased the extent of fibrosis in *Pkd1*^*RC/RC*^ mouse kidneys as measured both by picrosirius red staining and the levels of collagen-I protein, confirming our previous findings. Notably, however, the *Pkd1*^*RC/RC*^*Cav1*^−/−^ kidneys had no change in fibrosis with ouabain administration as determined by either method (Fig. 5). Representative images of the picrosirius red staining are shown in Fig. 5A, and representative immunoblots for collagen-I are shown in Fig. 5C. These results indicate that ouabain’s effects on kidney fibrosis relies on the presence of caveolae in the cells.

### 3.6. Ouabain increases activation of ERK and Akt in Pkd1^RC/RC^ but not Pkd1^RC/RC^Cav1^−/−^ kidneys

**Fig. 6.**
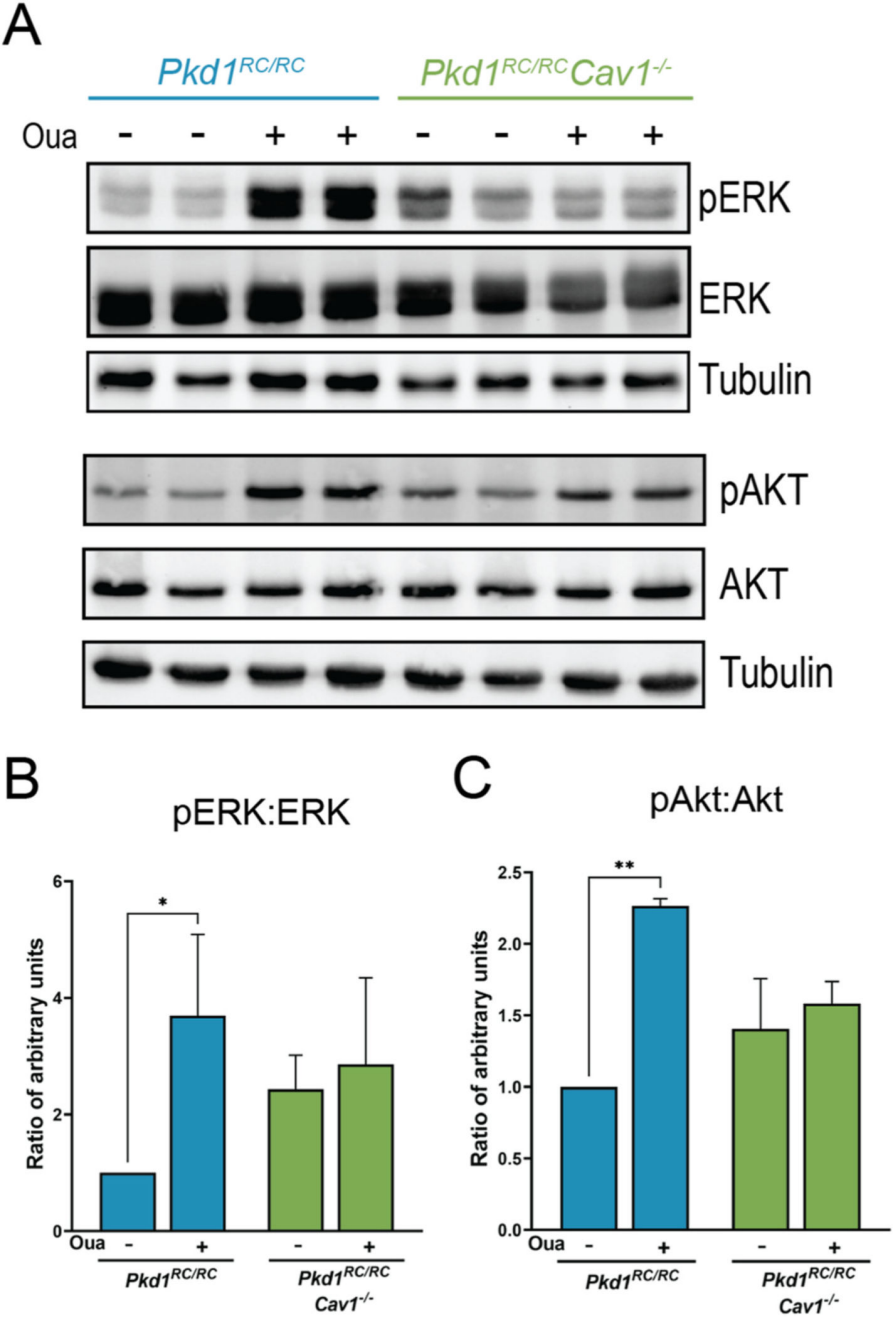
Ouabain-induced phosphorylation of ERK and Akt in *Pkd1*^*RC/RC*^ mouse kidneys is abolished in *Pkd1*^*RC/RC*^*Cav1*^−/−^ mice. (A–C) Ouabain increases the phosphorylation of ERK and Akt in *Pkd1*^*RC/RC*^ but not *Pkd1*^*RC/RC*^*Cav1*^−/−^ mice. Values are ±SEM from 3 animals per group, mixed male and female. *p < 0.05, **p < 0.01.

Ouabain-induced NKA signaling has been shown to activate different cell-signaling pathways, several of which overlap with pathways which are dysregulated in ADPKD [15,28,34,53]. In this study, we examined two key downstream NKA signaling mediators, the extracellular signal regulated kinase (ERK) and protein kinase B (Akt). As shown in Fig. 6A–C, despite the increased background levels of activated ERK and Akt in the *Pkd1*^*RC/RC*^ model, ouabain further augmented phosphorylation of both ERK and Akt [53,54]. This result supports the idea that ouabain worsens the ADPKD cystic phenotype through the NKA signaling pathway and its downstream effectors, ERK and Akt. In contrast, in the *Pkd1*^*RC/RC*^*Cav1*^−/−^ mouse, there is no change in the levels of ERK or Akt phosphorylation by ouabain administration.

## 4. Discussion

Our previous work has shown that ouabain increases cyst progression *in vitro* and *in vivo* and has provided evidence that this effect is mediated by a subpopulation of NKA with a higher-than-normal affinity for ouabain [19–22,55–57]. Here, we generated a new mouse model in which CAV1 is deleted on the background of a slowly progressive ADPKD model, the *Pkd1*^*RC/RC*^*Cav1*^−/−^ mouse, to show that ouabain’s pro-cystic effects depend on the presence of cell caveolae. Our main findings in this model include: 1) the disappearance of the high ouabain affinity that a subpopulation of NKA has in ADPKD; 2) the significant reduction in kidney cystic area and fibrosis to the basal levels of the *Pkd1*^*RC/RC*^ mouse model; 3) the decrease in ouabain-dependent cell proliferation that is a main driver of ADPKD cystogenesis [27]; and 4) lack of activation of ERK and Akt, two key mediators of NKA signaling induced by ouabain [21,27]. Altogether, these results support the idea that cell caveolae are required for the ouabain-induced increase in cyst development and NKA signaling in ADPKD.

Caveolae have been shown to participate in cell regulation through organization of different signaling platforms which harbor multiprotein complexes that transfer information from the cell surface to the cytoplasm and nucleus [39,58,59]. These cholesterol-rich structures have also been proposed as the subcellular location of the NKA signaling complex due to the interaction of CAV1 with a conserved caveolin-binding motif in NKA and the involvement of CAV1 in NKA signal transduction [37,38,60]. Within caveolae from myocardial cells, ouabain increases the association of NKA with CAV1, activates Src, and causes phosphorylation of the NKA-associated epidermal growth factor receptor (EGFR) [38,61]. These effects trigger Ca^2+^ oscillations, generate ROS, and activate signaling cascades that result in the activation of ERK, PKC, and Akt to induce cardiac cell hypertrophy [28,32,34, 62,63]. A similar mechanism has been shown to operate in cultured pig kidney LLCPK1 cells, with a loss of cell caveolae ablating the effects of ouabain [48].

Our results here support the involvement of CAV1 and caveolae in ouabain-induced and NKA-mediated signal transduction and cystogenesis in the *Pkd1*^*RC/RC*^ mouse model. Contrary to observations in *Pkd1*^*RC/RC*^ mice, physiological amounts of ouabain do not increase the total cystic area of *Pkd1*^*RC/RC*^*Cav1*^−/−^ mice (Fig. 3). In ADPKD kidneys, cyst growth is highly dependent on the abnormal increase in cell proliferation. We have previously shown that, in ADPKD cells, ouabain augments cell proliferation; here we present similar results in *Pkd1*^*RC/RC*^ mice, where a physiological dose of ouabain increases the number of Ki67-positive nuclei (Fig. 4) [55]. In *Pkd1*^*RC/RC*^*Cav1*^−/−^ mice, however, ouabain administration has no impact on the number of Ki67-positive nuclei, with both control and treated animals remaining at levels similar to those of *Pkd1*^*RC/RC*^ controls. This reduction in cell growth agrees with the measurements of cystic area of *Pkd1*^*RC/RC*^*Cav1*^−/−^ mice, which remained similar to *Pkd1*^*RC/RC*^ controls despite ouabain administration (Fig. 3). Therefore, caveolae are required for ouabain to cause an increase in cell proliferation and cystic growth in *Pkd1*^*RC/RC*^ mice. Interestingly, caveolar disruption did not result in a decrease in the basal cyst growth level of the *Pkd1*^*RC/RC*^ kidneys, despite the fact that caveolae are a center for signaling through different pathways. While the reason for this observation is presently unclear, it may depend on the complexity of ADPKD, in which many signaling pathways are contributing to the progression of the disease. Therefore, it is possible that other mechanisms independent from caveolae are involved in stimulating cyst growth. This is supported by studies showing that in ADPKD, there are abnormal intracellular calcium levels via the dysfunction of the primary cell cilium, or changes in the organization of components of the extracellular matrix, which activate ADPKD cystogenesis in a manner that would be independent of caveolae [2,64]. Additionally, it is possible that due to the slow progression of the disease in *Pkd1*^*RC/RC*^ mice (especially under the BL/6 background and during the 5 months span of our study), the increase in basal cystogenesis is relatively small and detection of changes might fall under the discrimination capacity of our assay. In any case, it is clear that from our results here caveolar deletion affects the pro-cystic effects of ouabain in *Pkd1*^*RC/RC*^ mice.

Another outcome of ouabain-induced NKA signaling is epithelial-to-mesenchymal transition (EMT) and tissue fibrosis, which has been seen in the heart, human ADPKD cells, and *Pkd1*^*RC/RC*^ kidneys [20,22,65]. In agreement with previous results, a relatively low dose of ouabain caused a significant increase in kidney fibrosis measured by collagen deposition and total collagen-I protein in *Pkd1*^*RC/RC*^ mice; however, in the *Pkd1*^*RC/RC*^*Cav1*^−/−^ mice, fibrosis was unchanged with ouabain administration (Fig. 5). Based on this result, it is clear that caveolae are essential for ouabain to cause kidney fibrosis in *Pkd1*^*RC/RC*^ mice as well.

A well-characterized downstream signaling event initiated by ouabain binding to NKA is the activation of Src which then causes an increase in the activation of both ERK and Akt, two NKA signaling mediators which are already hyperactivated in ADPKD [14,26,38,53]. We have previously shown that low doses of ouabain in human ADPKD cells increase the activation of the EGFR/Src/MEK/ERK pathway, though we have not previously explored the involvement of Akt in NKA signal transduction in ADPKD [21]. Due to the shared involvement of both mediators in ADPKD pathogenesis and NKA signaling, we studied the effects of ouabain on the phosphorylation levels of ERK and Akt in *Pkd1*^*RC/RC*^ and *Pkd1*^*RC/RC*^*Cav1*^−/−^ kidneys. As expected, ouabain significantly increased the activation of both ERK and Akt in *Pkd1*^*RC/RC*^ mouse kidneys, which likely enhances the cystic phenotype seen in this model. In the *Pkd1*^*RC/RC*^*Cav1*^−/−^ mouse, however, ouabain was unable to activate NKA signaling as shown by a lack of increase in the phosphorylation level of either protein with ouabain administration. Notably, however, the basal level of activation of both proteins is slightly higher than that of *Pkd1*^*RC/RC*^ controls (Fig. 6), suggesting that perhaps Src is more active in this model. The interaction of NKA with Src within caveolae maintains this kinase in an inactive state [40], therefore, it is possible that disruption of caveolae leads to an increase in basal Src activation and a slightly higher level of ERK and Akt phosphorylation. Interestingly, despite this, *Pkd1*^*RC/RC*^*Cav1*^−/−^ mice did not have a subsequent increase in cystic area, fibrosis, or cell proliferation compared with *Pkd1*^*RC/RC*^ controls.

While caveolae contribute to ouabain-induced NKA signaling and cystogenesis in ADPKD, we were surprised by the fact that removal of CAV1 also reverted the altered affinity of NKA for ouabain of the *Pkd1*^*RC/RC*^ kidneys to a value similar to that of wild type mice (Fig. 2) [22]. Therefore, in addition to being required for NKA downstream signaling capacity, our data support a role of caveolae on the ouabain affinity of NKA. It has previously been shown that the caveolar-specific NKA may have an increased ouabain affinity in the physiologic state, allowing the relatively ouabain resistant renal NKA to signal at the low circulating ouabain concentrations [66]. It is possible that under normal conditions, only a small fraction of NKA from caveolae have a high ouabain affinity, while the majority maintains the usual low ouabain affinity of the renal tissue. In the pathological setting of ADPKD, this high ouabain affinity NKA pool becomes a relatively larger portion of the total renal NKA, leading to hyperactivation of the NKA signaling cascade and the enhancement of cyst growth. In this scenario, caveolar disruption on the ADPKD background, by abolishing the highly sensitive NKA population (Fig. 2), lessens NKA signaling to prevent this pathway from significantly contributing to cyst progression. Currently, the mechanism or mechanisms underlying the change of NKA ouabain affinity in ADPKD is unknown. Our previous findings support the hypothesis that ouabain’s effects in ADPKD are due to an increase of NKA signaling secondary to the abnormally high ouabain affinity of NKA in ADPKD kidneys, as shown by the increase in ADPKD cyst growth when NKA is mutated to increase its affinity for ouabain [22]. We propose that interaction of NKA with specific proteins in the caveolar compartment of ADPKD cells may be responsible for the effect. Discovering the underlying factor (or factors) implicated will require additional experiments, which we are planning to pursue.

The presence of CAV1 in ADPKD cells has been previously reported, and we confirmed that the *Pkd1*^*RC/RC*^ mouse also contains caveolae located at the basolateral side of the renal colleting duct cells (Fig. 1) [55]. We also corroborated the finding by others that CAV1 knockout is sufficient to eliminate caveolar formation in the renal tubules in the *Pkd1*^*RC/RC*^*Cav1*^−/−^ mouse (Fig. 1) [49]. It is interesting that caveolar disruption seems to have little to no effect on ADPKD cyst progression as the amount of cystic area, kidney fibrosis, and cell proliferation that was independent of ouabain treatment were unchanged between *Pkd1*^*RC/RC*^ and *Pkd1*^*RC/RC*^*Cav1*^−/−^ controls (Figs. 3–5). Because *Pkd1*^*RC/RC*^ is a slowly progressive model of ADPKD which has preserved kidney health beyond 12 months of age, it is possible that the 5-month experimental time point that we used is still early to detect any impact of caveolar knockout alone on ADPKD progression [43]. However, it has been demonstrated that polycystin-1 does not localize to the caveolae, so it is possible that caveolar disruption does not affect the progression of ADPKD outside of the NKA signaling pathway [67].

## 5. Conclusions

In conclusion, our work here demonstrates that caveolae are necessary for the formation of the high ouabain affinity pool of NKA in ADPKD and supports the idea that ouabain-induced NKA signaling occurs primarily through the NKA signaling complex located within caveolae. While this work has advanced our understanding of the contributions of NKA signaling in ADPKD, more studies are needed to understand what caveolar alterations occur in ADPKD which influences NKA ouabain affinity. Future research should investigate potential changes within caveolae in the setting of ADPKD which may be influencing NKA ouabain affinity and whether this mechanism is targetable for ADPKD therapies.

## Data Availability

Data will be made available on request.
